# Antivirulence drugs against *Acinetobacter baumannii*: where do they stand?

**DOI:** 10.1007/s11274-025-04593-3

**Published:** 2025-10-30

**Authors:** Aya A. Elanany, Amany G. Khalifa, Ahmed S. Attia

**Affiliations:** 1 Department of Microbiology and Immunology, Faculty of Pharmacy, Mansoura University, Mansoura, 35516 Egypt; 2 Immunology & Regenerative Medicine Professional Master’s Program, Faculty of Pharmacy, Mansoura University, Mansoura, 35516 Egypt; 3 Department of Microbiology and Immunology, Faculty of Pharmacy, Cairo University, Kasr El-Ainy St, Room D-404, Cairo, 11562 Egypt

**Keywords:** Antivirulence, Acinetobacter baumannii, Monoclonal antibodies, Phage-derived enzymes, Resistance

## Abstract

*Acinetobacter baumannii* is a nosocomial Gram-negative pathogen of multi- and extensive-drug resistance phenotypes. In certain regions, multi-drug resistance rates can soar as high as 80–90%. Accordingly, reports by the World Health Organization and the Centers for Disease Control and Prevention underscore the urgent need for developing new antibiotics against *A. baumannii*. With the scarcity of novel antibiotics against this pathogen in the development pipeline, its rapid ability to develop and acquire resistance, and its versatile virulence weaponry, antivirulence therapeutics that can attenuate its virulence without adversely affecting growth may represent a promising treatment option with less selective pressure for resistance development. This review summarizes different antivirulence strategies against *A. baumannii*, which have been preclinically tested in various in vivo models. We also review different challenges and opportunities related to the realization of *A. baumannii* antivirulence drugs for clinical use.

## Background

Since the discovery of penicillin by Alexander Fleming in 1928, as many as 20 new classes of antibiotics were marketed by the 1970 s (Powers 2004; Laxminarayan et al. 2013). Since then, antimicrobials have improved human health and extended the normal human lifespan (Hutchings et al. 2019). However, antibiotic overuse and misuse over the years have led to the rapid selection of antibiotic-resistant bacteria and resistance gene transfer to other populations (Hutchings et al. 2019). Antimicrobial resistance (AMR) has become a serious concern for human health, as it is estimated to reach ~ 10 million deaths by the year 2050 (O’Neill 2016). In addition, critical medical procedures such as organ transplantation, cancer chemotherapy, and surgery are also compromised (Prestinaci et al. 2015). The World Health Organization (WHO) listed AMR as one of the top ten worldwide health issues that are worth tracking in the current decade (World Health Organization 2020).

*Acinetobacter baumannii* is a Gram-negative bacterium that has earned increasing attention as a significant opportunistic pathogen in healthcare settings worldwide. Despite being initially perceived as a relatively harmless organism, several studies delineated the virulence factors of *A. baumannii* (Harding et al. 2018; Kumar et al. 2021; Dolma et al. 2022; Shadan et al. 2023; Yao et al. 2023; Zhou et al. 2023; Lucidi et al. 2024). It is now considered a formidable threat primarily targeting critically ill patients. In the United States and Europe, *A. baumannii* infections constituted approximately 2% of all healthcare-associated infections, whereas rates were notably higher, around twice as much, in Asia and the Middle East (Reddy et al. 2010; Magill et al. 2014). In a more recent surveillance report with data received from 48 countries across 6 global regions, *A. baumannii* comprised 0.7% and 4.6% of all the isolated aerobic, facultative, Gram-negative bacilli in North America and the Middle East, respectively (Lob et al. 2016). In the European Economic Area, *Acinetobacter* spp. were among the most commonly reported bacterial species, with more isolates reported in 2021 than in 2020 across all countries (European Centre for Disease Prevention and Control and World Health Organization 2023). *A. baumannii* is implicated in a spectrum of nosocomial infections spanning diverse anatomical sites. Predominantly, infections attributed to *A. baumannii* manifest as ventilator-associated pneumonia or central-line-associated bloodstream infections (Weiner et al. 2016). *A. baumannii* infections tend to occur primarily in immunocompromised individuals with underlying comorbidities (Dexter et al. 2015).

The ability of *A. baumannii* to rapidly acquire resistance to multiple antimicrobial agents is attributable to an array of resistance mechanisms and has escalated its impact on patient outcomes and healthcare systems (Rolain et al. 2013; Giammanco et al. 2017). Antibiotic resistance mechanisms in *A. baumannii* include efflux pumps, drug-modifying enzymes, and modification of drug targets, among others (Shi et al. 2024). Furthermore, *A. baumannii*, by being naturally transformable and having a high genomic plasticity, facilitated both the acquisition and accumulation of multiple resistance determinants (McCarthy et al. 2021). Resistance to carbapenem, a last-resort antibiotic for multidrug-resistant (MDR) bacteria, was as high as 50% or above in *Acinetobacter* spp. isolated from southern and eastern countries in the WHO European Region (European Centre for Disease Prevention and Control and WHO Regional Office for Europe 2024). In Latin America and the Mediterranean region, MDR rates soar to as high as 80–90%, significantly surpassing those of other notable pathogens like *Pseudomonas aeruginosa* and *Klebsiella pneumoniae* (Giammanco et al. 2017; Ma and McClean 2021; Murray et al. 2022; Elwakil et al. 2023). These alarming MDR rates have prompted the Centers for Disease Control and Prevention (CDC) to categorize MDR *Acinetobacter* spp. as a serious threat, necessitating ongoing public health surveillance and prevention efforts (Centers for Disease Control and Prevention 2019). Additionally, the WHO has categorized carbapenem-resistant *A. baumannii* (CRAB) as a critical pathogen, underscoring the urgent need for research and development of new antimicrobial therapies (World Health Organization 2024).

Conventional antibiotics affect targets essential for bacterial survival, including cell wall synthesis, protein synthesis, and DNA replication, thereby exerting selective pressure, driving bacterial evolution, and leading to the emergence of antibiotic-resistant strains (Hotinger et al. 2021). Parent molecules of classical antibiotic classes were identified by screening natural products or chemical libraries for their bactericidal or bacteriostatic activity (Cegelski et al. 2008). Therapeutic strategies based on an antivirulence approach have emerged as an alternative to antibiotics. Virulence is the capacity of a microorganism to cause disease. Virulence factors are how bacteria establish infections in their host (Lee et al. 2003; Abd El-Aleam et al. 2021). They help bacteria colonize, evade immune defenses, suppress immunity, obtain nutrients, and attack host cells (Sabino et al. 2023). Unlike traditional antibiotic targets, most virulence traits are not essential for bacterial survival (Allen et al. 2014). An antivirulence agent targets bacterial virulence without a bacteriostatic or bactericidal effect (Maura et al. 2016; Hotinger et al. 2021). By this definition, antivirulence therapeutics are a proposed alternative to classical antibiotics with the advantage of the expected lower evolutionary pressure (Clatworthy et al. 2007; Baron 2010; Ogawara 2021). Antivirulence drugs are thought to help the host immune system prevent bacterial colonization or clear established infections in immunocompetent hosts, or they can be synergistic with antibiotics in immunocompromised individuals (Rasko and Sperandio 2010; Cascioferro et al. 2014; Rezzoagli et al. 2020).

Several other attributes support the case for developing antivirulence therapeutics. Since antivirulence drugs target specific pathogen-host interaction events, they would, by definition, be limited to human use, thereby further decreasing the risk of resistance that can arise to clinical antibiotics in non-human environments such as the livestock industry (Escaich 2008; Laxminarayan et al. 2013). Moreover, virulence targets are essential only in the context of infection, so excreted antivirulence drugs and/or their metabolites are unlikely to select for *A. baumannii*-resistant mutants in non-human environmental niches (Russo et al. 2016). An additional advantage is the fewer adverse effects exerted on the host’s beneficial microbiota (Cegelski et al. 2008; Hotinger et al. 2021). Besides, antivirulence drugs are expected to be safe since virulence pathways and factors are exclusive to pathogens and not present in mammalian cells (Calvert et al. 2018).

In this review, we highlight antivirulence agents developed for *A. baumannii*, with the condition of being tested in an in vivo model. We have excluded therapeutics targeting quorum sensing (QS), biofilm formation, and iron acquisition since other groups have reviewed these (López-Rojas et al. 2013; Tay and Yew 2013; Fleitas Martinez et al. 2019; Bamunuarachchi et al. 2021; Zhong and He 2021; Law and Tan 2022; Zeng et al. 2023). Finally, we discuss the challenges and opportunities of developing drugs targeting the virulence arsenal of *A. baumannii*.

## Antivirulence agents tested in vivo against *A. baumannii*

### Synthetic compounds

Lipopolysaccharide (LPS) is a virulence factor of *A. baumannii* for which different antivirulence interventions were tested. The shedding of LPS during the growth of *A. baumannii* and the subsequent development of the toll-like receptor (TLR)−4-mediated inflammation in a lethal bloodstream infection model highlighted the role of LPS in the *A. baumannii* virulence (Lin et al. 2012). Accordingly, Lin and co-workers investigated the potential therapeutic role of LpxC-1, a small molecule inhibitor of LpxC, an enzyme involved in LPS lipid A synthesis. LpxC-1 diminished the TLR-4 binding potency of LPS when incubated with LPS derived from the culture supernatants or extracted from the cells. In an in vivo model of bloodstream infection, LpxC-1 completely rescued both immunocompetent and neutropenic mice from an otherwise lethal infection when administered post-infection (Lin et al. 2012). LpxC-1-treated immunocompetent mice had significantly lower blood and tissue (liver, spleen, and lung) bacterial burdens, lower serum LPS, and lower inflammatory cytokine levels (Lin et al. 2012). In addition, LpxC-1 enhanced the RAW macrophage-mediated killing of bacteria (Lin et al. 2012).

Another approach to target LPS is to trap it using a high molecular weight polymer. Phosphorylated high-molecular-weight polyethylene glycol 15–20 (Pi-PEG 15–20) was tested as a potential antivirulence agent against different peritoneal sepsis-causing pathogens, including the standard carbapenem-resistant *A. baumannii* ATCC 19606 strain. Pi-PEG 15–20 could trap LPS and inhibit extracellular LPS-induced interleukin (IL)−8 production by the human intestinal epithelial cell line HT-29. However, the unphosphorylated polymer exhibited a similar effect. When *Caenorhabditis elegans* was challenged with *A. baumannii* ATCC 19606, Pi-PEG 15–20 significantly reduced mortality compared to the untreated group (Zaborin et al. 2014).

Another virulence trait of *A. baumannii* is its ability to evade innate immunity (Lin et al. 2012; Bruhn et al. 2015; Wong et al. 2017). Consequently, using immunomodulators can be a viable antivirulence strategy. Lysophosphatidylcholine (LPC) is an immunomodulator involved in immune cell recruitment and activation, as well as bacterial elimination during infections (Lauber et al. 2003; Yan et al. 2004; Mesquita et al. 2008). Smani et al. (2015) reported the LPC efficacy in *A. baumannii* murine models of peritoneal sepsis and pneumonia, as it increased survival by 40% or more. LPC in combination with colistin, tigecycline, or imipenem improved bacterial clearance, reduced bacteremia, and improved survival in the tested models as compared to LPC or the antibiotic treatment alone (Parra Millán et al. 2016). Subsequently, the authors tested administering one or two LPC doses in combination with colistin to the same disease models using *A. baumannii* clinical strains (Miró-Canturri et al. 2021). The two-dose regimen led to a slightly higher bacterial clearance from the tested organs. LPC is thought to enhance neutrophil function in combating *A. baumannii* infection and the subsequent production of pro-inflammatory cytokines such as IL-1, IL-6, and tumor necrosis factor-alpha (TNF-α) (van Fassen et al. 2007; García-Patiño et al. 2017; Miró-Canturri et al. 2021). LPC can be viewed as an antivirulence intervention since it is an immunomodulator with proven in vivo activity of enhancing bacterial clearance.

Outer membrane proteins (Omps) are embedded in the *A. baumannii* outer membrane (OM) and play key roles in cellular permeability and virulence. One of the most important Omps in *A. baumannii* is OmpA (Choi and Lee 2019). It is a highly conserved porin involved in antibiotic and serum resistance, adherence, biofilm formation, cytotoxicity, and apoptosis (Morris et al. 2019; Zhou et al. 2023). AOA-2 is a cyclic hexapeptide potential binder and blocker of the *A. baumannii* OmpA without bactericidal activity. Vila-Farrés et al. (2017) first reported it as the hexapeptide achieving the highest inhibitory activity among six synthetic hexapeptides on *A. baumannii* adherence to A549 cells. When administered alone at a low dose of 10 mg/kg/day to mice challenged with *A. baumannii* ATCC 17978, the bacterial loads in the liver and spleen were reduced by about four-log cycles lower than that of the untreated group (Vila-Farrés et al. 2017). Besides, bacterial loads in blood and mortality rates were about 70% lower in the AOA-2-treated group (Vila-Farrés et al. 2017). In addition, AOA-2 and colistin synergize against *A. baumannii *in vitro and in the murine peritoneal sepsis model. Combining AOA-2 and colistin reduced the bacterial load in the spleen and lungs by about two-log cycles, exceeding the therapeutic effect of colistin alone. Mice receiving combined treatment with AOA-2 plus colistin showed a greater increase in survival and lack of bacteremia compared with those receiving colistin alone or the controls. These results suggest that the AOA-2 and colistin combination can be a promising treatment option for *A. baumannii* infections (Parra-Millán et al. 2018).

Another virulence tool crucial for successful *A. baumannii* infections is its extracellular enzymes, which represent a good pool for antivirulence drug discovery. For instance, phospholipase C was effectively inhibited by miltefosine. Phospholipase C is an enzyme produced by *A. baumannii* and contributes to its virulence by causing cytolysis of host cells as well as hemolysis (Dorlo et al. 2012). Miltefosine is a phosphatidylcholine analog belonging to the class of alkylphosphocholine drugs. Miltefosine effectively reduced the *A. baumannii* hemolytic and cytolytic activity against horse red blood cells (Fiester et al. 2019). Additionally, miltefosine treatment protected A549 human alveolar epithelial cells from damage caused by all the tested *A. baumannii* strains (Fiester et al. 2019). The most effective miltefosine treatment was as low as 12 µM, which was determined based on its effectiveness in preventing the breakdown of red blood cells (Fiester et al. 2019). In an animal model, miltefosine treatment significantly decreased mortality in *Galleria mellonella* infected with *A. baumannii* ATCC 19606 (Fiester et al. 2019).

Notably, miltefosine had different effects on bacterial growth in the tested bacterial strains. While it did not affect the *A. baumannii* 3494 growth, it significantly reduced the *A. baumannii* ATCC 19606 growth and significantly enhanced the AB5075 growth at 20 h of incubation (Fiester et al. 2019). In the latter case, miltefosine was found not to have been utilized by the bacteria for carbon when supplemented as the sole carbon source (Fiester et al. 2019). This indicates that while miltefosine has the potential for treating *A. baumannii* infections owing to its antivirulence effect, further studies are still needed to investigate the mechanisms of its strain-specific variable effect on bacterial growth (Fiester et al. 2019). Should this phenomenon be of biological significance, both improved and halted bacterial growth may detract from the clinical application of miltefosine as an antivirulence drug. Enhanced growth would pose a greater challenge for the host immune system, and adversely affecting bacterial growth would exert an evolutionary pressure for the development of resistance, violating the concept of antivirulence therapy. That said, miltefosine was clinically tested for leishmaniosis (Knight Therapeutics Inc 2010). At least regarding safety considerations, miltefosine, having proven efficacy in another infectious disease, may not have a high commercial risk of clinical translation as an antivirulence drug against *A. baumannii*.

Synthetic macrocycles designed to interact with certain virulence agents may also be promising. Pillar[5]arene (P[5]a) is a novel macrocycle identified to be the most promising molecule with the highest affinity to different homoserine lactones (HSLs), which are QS molecules. P[5]a is a bifunctional agent where its hydrophobic core quenches HSLs with binding affinity dependent on the length and hydrophobicity of the acyl chain, and its periphery interacts with LPS via electrostatic attraction (Jonkergouw et al. 2023). Although the only effect of P[5]a against *A. baumannii* in this study was the biofilm inhibition in clinical MDR *A. baumannii* isolates, other antivirulence effects were reported in *P. aeruginosa*. The minimum inhibitory concentrations (MICs) of four tested antibiotics with intracellular targets, amikacin, cefepime, ceftazidime, and meropenem, were lower when combined with P[5]a against six MDR *P. aeruginosa* clinical isolates as compared to the antibiotic alone. This indicates that the interaction of P[5]a with LPS may adversely affect bacterial membrane integrity and enhance the penetration of conventional antibiotics. When tested in vivo in mice challenged with intranasal administration of LPS, the analyzed inflammatory cytokines were significantly lower after 24 h in the P[5]a-treated group (Jonkergouw et al. 2023).

Nutrient metabolites associated with low-virulence phenotypes may be further investigated as virulence-attenuating agents. One example is L-serine, a non-toxic nutrient metabolite that could reverse *A. baumannii* virulence as observed upon conducting a comparative metabolomic analysis (Zhou et al. 2024). When tested in vivo, L-serine improved *G. mellonella* survival in 8 of the 10 highly virulent clinical *A. baumannii* strains used to induce infection. Mechanistically, L-serine was found to reduce virulence by upregulating the NAD-dependent deacetylase sirtuin-1 (SIRT1) in *A. baumannii*-infected epithelial Beas 2B cells in a dose-dependent manner (Zhou et al. 2024). Furthermore, L-serine reduced *A. baumannii*-induced reactive oxygen species (ROS) and mitochondrial ROS by about 50% and reduced mitochondrial dysfunction in Beas 2B cells in a SIRT1-dependent manner (Zhou et al. 2024). In addition, the NOD-, LRR- and pyrin domain-containing protein 3 (NLRP3) inflammasome and its downstream effectors, IL-1ß and IL-18, were repressed on the protein and mRNA levels in L-serine-treated cells (Zhou et al. 2024). NLRP3 activation and IL-1ß and IL-18 release are involved in *A. baumannii* pathogenesis in pulmonary infections (Dikshit et al. 2018),

The argument for antivirulence drugs calls for innovative approaches, such as the repurposing of FDA-approved drugs whose primary indications might not be infectious diseases. Since *A. baumannii* degrades phenylacetic acid (PAA) under stress, it was shown that diclofenac, a PAA derivative approved by the FDA as a non-steroidal anti-inflammatory drug, can attenuate the virulence of *A. baumannii* (Hooppaw Anna et al. 2022; Bisaro et al. 2024). While it did not negatively impact bacterial growth when administered alone to clinical *A. baumannii* isolates, diclofenac was synergistic to colistin against a colistin-resistant strain. Employing whole-cell transcriptomic and proteomic analyses, synergy was mediated by the induction of oxidative stress and downregulation of the type IV pili. The in vivo experiments agreed with the synergism observed in vitro (Bisaro et al. 2024). In an acute murine pneumonia model, neither colistin nor diclofenac significantly affected bacterial burdens when mice were challenged with a colistin-resistant strain. However, the combined treatment reduced the bacterial loads by at least three-log cycles, depending on the organ tested.

### Compounds of natural origin

Similar to the discovery of early antibiotics, natural products may still offer valuable antivirulence compounds. A study by Guo et al. (2021) reported that capsaicin exhibited dose-dependent synergy with colistin, where bacterial cells have regained or improved sensitivity to colistin by dropping the MIC value below the susceptibility breakpoint. The possible mechanisms of capsaicin antivirulence were investigated in vitro, where capsaicin-treated bacterial cells had increased outer membrane permeability in a concentration-dependent manner, while the inner membrane was not affected. The authors argued that this may explain the mechanism of the synergistic action of capsaicin with colistin, where the former may enhance the penetration of the latter so it can act on LPS. Other antivirulence mechanisms of capsaicin included the inhibition of biofilm formation and the reduction of intracellular ATP levels in all the tested strains. The capsaicin/colistin combination was tested in vivo in male BALB/c mice, where bacteremia was induced using an *A. baumannii* ATCC 19606 variant exhibiting a lab-induced colistin-resistant phenotype. Bacterial loads in all the tested organs: liver, lung, kidney, and spleen, were significantly lower in the combination-treated group as compared to those in any of the two agents alone (Guo et al. 2021).

Chrysin is a naturally occurring flavonoid compound with proven in vivo efficacy against *A. baumannii*. Chrysin was found to exhibit synergy with colistin whereby it decreased colistin MIC to values lower than the susceptibility breakpoint by increasing membrane permeability and enhancing the membrane-destroying effect of colistin (Zhao et al. 2022). Most importantly, when tested in vivo, the chrysin/colistin combination decreased the case fatality rate by 90% in *G. mellonella* larvae infected with a colistin-resistant clinical *A. baumannii* isolate (Zhao et al. 2022). Carried further into a vertebrate infection model, the combination treatment results confirmed the invertebrate model observations since a lower bacterial load was recovered from the infected thigh muscles of mice receiving chrysin and colistin as compared to that in any of the individual interventions alone (Zhao et al. 2022). In addition, the non-toxic effect of a wide range of sub-MIC concentrations of chrysin on the viability of murine macrophage cells (RAW 264.7 cells) supports the potential of chrysin as an adjunct antivirulence drug.

Pyrogallol is another natural product that was found to inhibit *A. baumannii* virulence without affecting bacterial growth (Abirami et al. 2021). When carried further into mechanistic and in vivo studies, pyrogallol was found to induce several-fold inhibition of different virulence-associated protein expression, including OmpA, protease, catalase, and antioxidant defense proteins such as superoxide dismutase and catalase (Abirami et al. 2023). In a zebrafish infection model, about 90% of animals receiving pyrogallol at a low dose of 20 µg/mL survived for 3.5 days post-infection, while all untreated animals died by the same period. Quantifying the bacterial burdens in the sacrificed animals showed a significant reduction of about five-log cycles in the pyrogallol-treated group (Abirami et al. 2023). In addition, treated animal sera were analyzed for non-specific immune response markers. Pyrogallol treatment was found to improve serum myeloperoxidase and lysozyme activity as well as leukocyte respiratory burst activity (Abirami et al. 2023).

### Phage-derived enzymes

In contrast to using bacteriophage-based therapy, which carries the risk of replication ability that may deter most clinicians and the public from their use, phage-derived enzymes lack such risk, which makes them a more acceptable therapeutic option (Verbeken et al. 2014; Chen et al. 2022). For example, Dpo48, a capsule depolymerase encoded by *A. baumannii* phage IME200, was studied in a *G. mellonella* infection model and a murine peritoneal sepsis model. In *G. mellonella*, survival rates were higher for the group infected with Dpo48-pretreated bacteria. In the mammalian model, post-inoculation treatment with Dpo48 resulted in significantly fewer bacterial loads in blood and the tested organs: liver, lung, kidney, and spleen, and a 100% survival rate was recorded. Toxicity was assessed in vivo in mice, and there was no significant difference in blood biochemical analyses and histopathological organ examination between the control and enzyme-treated animal groups (Liu et al. 2019).

Another bacteriophage-derived depolymerase is the K2 capsule-specific depolymerase. The enzyme was specific in affecting only *A. baumannii* strains of the K2 capsular type, which is consistent with the spectrum of its parent phage. In a 24-h in vitro experiment, enzyme-treated isolates remained as sensitive to the enzyme as the control-treated isolates, which indicated a low likelihood of developing resistance. It decreased the mortality rate of *G. mellonella* larvae when administered either before (enzyme-pretreated bacteria) or after the bacterial challenge with *A. baumannii* NIPH 2061, without affecting bacterial viability. Furthermore, the enzyme improved survival rates in immunosuppressed male and female BALB/c mice with septicemia induced by the same bacterial strain. Inflammatory markers, including TNF-α and IL-6, were significantly lower in the enzyme-treated mice as compared to those in the mock-treated ones. The authors argued that the bacterial killing effect is mediated by the lytic effect of the host complement system since mice were initially immunocompromised using cyclophosphamide which does not affect leukocytes involved in complement response. This was validated by an in vitro experiment using human serum where depolymerase treatment sensitized bacteria to serum-killing (Oliveira et al. 2019).

Additionally, Dp49, another phage-derived capsule depolymerase, improved survival rates in a murine peritoneal sepsis model. All enzyme-treated mice survived during the 96-h experimental period, and bacterial loads in the homogenate of the tested organs: liver, spleen, and lung, were significantly lower by more than four-log cycles. However, mice infected with the phage-sensitive strain and treated with the phage had markedly lower bacterial counts than the enzyme-treated ones. The enzyme, per se, did not exert any bactericidal activity since the bacterial counts of the sensitive strains remained unchanged in Dp49-treated inactivated serum, but bacterial counts were significantly lower in active serum with Dp49 (Wang et al. 2020).

Dpo71 is another phage-derived polysaccharide depolymerase that was tested in vivo in *G. mellonella*. The Dpo71/colistin co-treatment showed the highest survival percentage in *G. mellonella* challenged with an MDR *A. baumannii* clinical strain as compared to monotherapy with either agent. Dpo71 could act as an adjuvant to colistin at half the MIC value of the latter in vitro in serum inoculated with the bacteria. The Dpo71/colistin combination led to almost complete eradication of bacteria, while colistin alone did not affect bacterial counts at the same serum concentration. The enzyme did not exhibit an antibacterial effect in inactivated serum mixed with two different *A. baumannii* strains sensitive to the parent phage. In accordance, an eight-log reduction in bacterial counts was recorded for an active serum with sensitive bacteria treated with the enzyme, which indicated that Dpo71 sensitizes the bacteria to serum-killing. The possible mechanisms of action elucidated for Dpo71 in this study are its biofilm inhibitory effect, in a dose-dependent manner, and a pre-formed biofilm-disrupting effect. Dpo71 did not independently affect OM stability but improved the destabilizing activity of colistin to the OM when combined with it (Chen et al. 2022). However, it is worth noting that all the mechanistic studies were performed in vitro.

### Monoclonal antibodies

Since antibodies are a natural mechanism of defense against infectious diseases, monoclonal antibodies (mAbs) can be artificially supplemented as a treatment option. Indeed, there are 10 mAbs licensed for viral infections and bacterial toxins (Casadevall and Paneth 2024). In the case of *A. baumannii*, with its concerning drug resistance profile and urgent need for novel therapeutics, mAbs can be a viable treatment option, especially with the use of mAb cocktails to target this antigenically diverse pathogen.

13D6 is a mAb directed against the *A. baumannii* K1 capsular polysaccharide (Russo. et al. 2010). Opsonization by 13D6 increased the neutrophil-mediated bactericidal activity in the tested K1-positive *A. baumannii* strains in vitro by two to four times, depending on the strain, compared to treatment with neutrophils in the absence of the antibody. When tested in vivo, 13D6 increased bacterial clearance in a rat soft tissue infection model by about four-log cycles compared to treatment with the vehicle control. However, the in vivo tested K1-negative strain, AB979, was not affected by 13D6 treatment, further confirming the antibody target (Russo et al. 2013). Murine mAbs must first be humanized for use in humans (Buchhorn de Freitas and Hartwig 2022). This is why the next step for carrying 13D6 into clinical practice would be its humanization.

C8 is another mAb that binds *A. baumannii* capsular carbohydrate and enhances opsonophagocytosis. C8 improved survival in C3HeB/Fe mice challenged with a clinical *A. baumannii* strain to induce sepsis (Nielsen et al. 2017). An aspiration pneumonia model was also established via oropharyngeal aspiration with the same bacterial strain. In the bloodstream infection model, immediate IV administration of 5 µg C8 led to complete protection against the infection compared to the isotype control mAb. To test the effect of delaying C8 administration, the authors administered it intraperitoneally (IP) at a dose of 50 µg at two different time points post-infection. When treated 30 min after the bacterial challenge, mice were completely protected. In the aspiration pneumonia model, C8 was also effective in almost complete protection against infection when administered at the same small dose of 5 µg. Complete protection was achieved upon using IP treatment with 50 $\mu$g C8 either immediately or four hours post-infection as compared to the isotype mAb control. Bacterial loads in the lung homogenate and blood of C8-treated mice were significantly lower in comparison with those in the control mice. In the bloodstream infection model, C8 was synergistic with colistin since the combination treatment resulted in 90% survival improvement, while colistin alone was completely ineffective. Interestingly, no escape mutants that would evade C8 binding developed when *A. baumannii* was serially passaged in complement-active CD-1 mouse serum in the presence of C8 or the isotype control. A fully humanized variant of C8 was developed by the authors in collaboration with an industry partner and retained its bacterial surface binding ability. The humanized clone resulted in superior enhancement of phagocytosis by human macrophages as compared to that of the murine mAb. Humanized C8 completely protected mice against IV infection (Nielsen et al. 2017).

Another humanized monoclonal anti-*A. baumannii* antibody is mAb 65, which was developed using hybridoma technology by immunizing mice with 2 extensively drug-resistant clinical *A. baumannii* strains to which the initial antibody developed by the group, C8 described above, did not bind. The mAb 65 functioned as an opsonin, improving internalization into murine macrophages by about 10-fold in the presence of both complement-active and heat-inactive CD-1 mouse serum. In a murine bloodstream infection model using a clinical *A. baumannii* strain (VA-Ab41), 75% of mice treated with 5 µg of mAb 65 survived the infection. To reflect the clinical treatment scenario where there is likely to be a delay in treatment onset, the authors administered mAb 65 IP 30 min or 1 h after the bacterial challenge. All mice survived at a mAb amount of 100 µg, while all mice treated with the isotype antibody control died. This was confirmed by a 75% reduction in blood bacterial burden in the mAb-treated group. In an aspiration pneumonia model, mAb 65 treatment rescued about 70% of mice after four days post-infection, while all mice receiving the isotype antibody control died after two days. Nearly a complete bacterial clearance from the lungs and blood of the mAb 65-treated mice was achieved two days after the bacterial challenge. When a small dose of mAb 65 was used in combination with colistin, all mice intravenously infected with *A. baumannii* survived, while more than half of the mice receiving either of the two interventions alone died. Interestingly, mAb 65 binding was not affected after serial passaging of VA-Ab41 20 times in the presence of mAb 65, indicating that no escape mutants emerged. This was reflected in the maintenance of the in vivo efficacy of mAb 65 since it could still completely rescue mice challenged with VA-Ab41 serially passaged in the presence of the mAb 65 (Nielsen et al. 2021).

The same group later developed a bispecific monoclonal antibody (bsmAb) called C73, which combined and exceeded the strain coverage of both previously developed mAbs: C8 and mAb 65, when tested against a panel of > 300 *A. baumannii* clinical isolates (Nielsen et al. 2023). C73 enhanced human macrophage uptake of extensively drug-resistant (XDR) clinical *A. baumannii* isolates, which were resistant to phagocytosis without mAb opsonization (Nielsen et al. 2023). C73 opsonization activity was higher than any of the two mAbs alone and was comparable to or exceeded incubation in individual mAb combinations, depending on the strain (Nielsen et al. 2023). Moreover, mice were protected from a lethal bloodstream infection induced by the three susceptible *A. baumannii* strains tested when treated with C73, at a survival rate higher than any of the two mAbs alone or combined. When combined with colistin, a small dose of C73 completely rescued mice from lethal bacteremia, indicating its synergistic activity with colistin, since mice receiving any of the two agents alone died (Nielsen et al. 2023).

The same group also developed another mAb with broad strain coverage by immunizing mice with a cocktail of four *A. baumannii* clinical isolates to which the bsmAb C73 did not bind (Slarve et al. 2023). The resulting mAb was designated mAb 5 and was humanized by an industrial entity (Slarve et al. 2023). As compared to C73, mAb 5 could bind to about 25% more isolates of the tested panel of 650 isolates from domestic and international sources, with each of the mAbs having a distinct strain coverage pattern (Slarve et al. 2023). Humanized mAb 5 completely rescued mice from an otherwise lethal bloodstream infection induced by two different clinical *A. baumannii* isolates (Slarve et al. 2023).

The most recent addition to the group’s arsenal of mAbs was mAb 10, developed by immunizing mice with 30 strains to which none of the previously developed mAbs could bind (Slarve et al. 2024). The overall strain coverage of the previously reported mAb cocktail was enhanced by about 10% by the addition of mAb 10, reaching about 72% of the tested 550 strains obtained from national and international sources (Slarve et al. 2024). When tested in vivo, mAb 10, starting from as low as 5 µg or above, rescued all mice from a lethal bloodstream bacterial infection (Slarve et al. 2024). A small dose of the humanized mAb 10 also rescued all mice in the bloodstream infection model. In a rat soft tissue infection model, the abscess fluid of mAb 10-treated mice had almost no detectable bacterial burdens by 72 h post-infection as compared to mice receiving the isotype antibody control (Slarve et al. 2024).

To summarize, Table 1 provides a comprehensive overview of the reported antivirulence agents against *A. baumannii*, categorized by their targeted virulence factor(s) and the in vivo model(s) employed for evaluation. The in vivo studies summarized in Table 1 utilize a wide array of experimental models, including immunocompromised and immunocompetent murine systems, invertebrate models such as *G. mellonella* and *C. elegans*, and non-murine vertebrate models like zebrafish. These models not only validate the efficacy of antivirulence agents but also provide insights into their mechanistic action under physiologically relevant conditions. Moreover, some studies tested antivirulence/antibiotic combinations against *A. baumannii* infections. This further supports the promise of these antivirulence interventions, especially since they were combined with colistin, the standard antibiotic treatment for XDR *A. baumannii* infections (Nielsen et al. 2017).

**Table 1:** Antivirulence agents with proven in vivo efficacy against *A. baumannii*

Antivirulence agent			Virulence factor(s) targeted	In vivo model deployed	Bacterial strain(s)	Reference
**Antivirulence agents tested in immunocompromised murine models:**						
**LpxC-1**			LPS synthesis	C3H/FeJ mice & neutropenic BALB/c mice (bloodstream infection)	XDR clinical strain	(Lin et al. 2012)
**Dpo48**			Capsule	Normal & immunocompromised BALB/c female mice (peritoneal sepsis) & *G. mellonella*,	Colistin-resistant clinical strain	(Liu et al. 2019)
** K2 depolymerase**			Capsule	Immunosuppressed male & female BALB/c mice (peritoneal sepsis) & *G. mellonella*	K2 capsular type NIPH 2061 strain	(Oliveira et al. 2019)
**Chrysin** (+ colistin)			Biofilm (in only some of the tested strains), outer membrane integrity	Neutropenic female ICR mice (thigh infection model) & *G. mellonella*	Colistin-resistant clinical strain	(Zhao et al. 2022)
**Antivirulence agents tested in normal murine models**:						
**13D6**			Capsular K antigen 1	Male Long-Evans rats (soft tissue infection)	AB307-0294 (K1 positive) & a clinical K1-negative strain	(Russo et al. 2013)
**LPC**			Immune evasion	Female C57BL/6 mice (peritoneal sepsis & pneumonia)	ATCC 17978 and clinical strains	(Smani et al. 2015; Parra Millán et al. 2016; Miró-Canturri et al. 2021)
**C8**		alone	A capsular carbohydrate moiety (specific target unidentified)	Male C3HeB/Fe mice (bloodstream infection & aspiration pneumonia)	XDR clinical strains	(Nielsen et al. 2017)
		+ colistin		Male C3HeB/Fe mice (bloodstream infection)		
**AOA-2**	alone		OmpA	Female C57BL/6 mice (peritoneal sepsis)	ATCC 17978	(Vila-Farrés et al. 2017)
	+ colistin				PDR clinical strain	(Parra-Millán et al. 2018)
**Dp49**			Capsule	Female BALB/c mice (peritoneal sepsis)	Clinical strains	(Wang et al. 2020)
**mAb 65**		alone	A capsular carbohydrate moiety (specific target unidentified)	C3HeB/Fe mice (bloodstream infection & aspiration pneumonia)	XDR clinical strain	(Nielsen et al. 2021)
		+ colistin		C3HeB/Fe mice (bloodstream infection)		
**Capsaicin** (+ colistin)			Biofilm, outer membrane integrity	Male BALB/c mice (bacteremia)	Colistin-resistant ATCC 19606	(Guo et al. 2021)
**C73**		alone	Capsular K antigens 9 & 22 (target predicted bioinformatically)	C3HeB/Fe mice (bloodstream infection & aspiration pneumonia)	XDR clinical strains	(Nielsen et al. 2023)
		+ colistin		C3HeB/Fe mice (bloodstream infection)		
**mAb 5**			Capsular O antigen 1 (target predicted bioinformatically)	Male C3HeB/Fe mice (bloodstream infection)	Clinical strains	(Slarve et al. 2023)
**P[5]a**			Biofilm, LPS	Female C57BL/6 J mice	LPS derived from *P. aeruginosa*	(Jonkergouw et al. 2023)
**mAb 10**			Capsular K antigens 2 & 23 (target predicted bioinformatically)	C3HeB/Fe mice (bloodstream infection) & Sprague-Dawley rats (soft tissue wound infection)	Clinical strains	(Slarve et al. 2024)
** Diclofenac ** (+ colistin)			Oxidative stress, type IV pili	Female C57BL/6 mice (pneumonia)	Colistin-resistant strain	(Bisaro et al. 2024)
**Antivirulence agents tested in non-murine vertebrate models**:						
** Pyrogallol**			Different virulence-related proteins	Zebrafish	MTCC9829	(Abirami et al. 2023)
**Antivirulence agents tested in invertebrate models**:						
**Pi-PEG 15–20**			Biofilm, LPS	*C. elegans*	ATCC 19606	(Zaborin et al. 2014)
**Miltefosine**			Phospholipase C	*G. mellonella*	ATCC 19606	(Fiester et al. 2019)
**Dpo71** (+ colistin)			Capsule, biofilm	*G. mellonella*	MDR clinical strain	(Chen et al. 2022)
**L-Serine**			SIRT1	*G. mellonella*	Clinical strains	(Zhou et al. 2024)

*LPC *lysophosphatidylcholine, *mAbs *monoclonal antibodies, *MDR *multidrug-resistant, *SIRT1 *NAD-dependent deacetylase sirtuin-1, *PAN *pandrug-resistant, *Pi-PEG 15–20 *phosphorylated high-molecular-weight polyethylene glycol 15–20, *P[5]a* pillar[5]arene, *XDR* extensively drug-resistant

### Antivirulence agents tested or currently undergoing testing clinically against *A. baumannii*

Upon using the ClinicalTrials.gov and the WHO International Clinical Trials Registry Platform (ICTRP) search portal, no clinical trials are ongoing or have been completed to test antivirulence agents against *A. baumannii* up to the end of the year 2024.

## Challenges in antivirulence drug development against *A. baumannii*

### Failure to fit the definition of an antivirulence agent

We argue, in accordance with other researchers (Allen et al. 2014; Lau et al. 2023), that an ideal antivirulence drug should not adversely impact pathogen growth; that is, to be devoid of any bacteriostatic/bactericidal effect (Rasko and Sperandio 2010). However, some agents fail to meet this definition, where their virulence-inhibiting effects are a means of their antibacterial action rather than an independent effect. This observation can be recorded in several antivirulence drug reports.

For example, compound 62520, a synthetic small pyrimidine-dione derivative, was found to inhibit the *ompA* gene expression and biofilm formation in the standard *A. baumannii* ATCC 17978 strain and most of the clinical CRAB isolates tested in a dose-dependent manner (Na et al. 2021). However, it exhibited bacteriostatic activity below the MIC (Na et al. 2021). Another example is the silver nanoparticles, which could downregulate the virulence-related genes in MDR *A. baumannii* isolates above their MIC concentration (Hetta et al. 2021).

Farshadzadeh et al. (2022) reported that the anti-biofilm activity of a dermcidin‑derived peptide (DCD‑1 L) was achieved at duplicates of the MIC. Moreover, curcumin-Nisin-based poly (L-lactic acid) nanoparticle-based photo-sonodynamic therapy had antibiofilm activity in vitro, which was further validated by a time-dependent reduction in biofilm-related genes. However, the preparation adversely affected planktonic cell growth (Pourhajibagher et al. 2022). A phage-derived capsule depolymerase was found to inhibit biofilm formation and disperse existing biofilms in a clinical *A. baumannii* isolate in vitro at amounts of 10, 50, and 100 ng but had a bactericidal effect mediated by cytoplasmic membrane destabilization, leading to cell lysis and death at 100 ng (Shahed-Al-Mahmud et al. 2021).

An antisense compound targeting the *lpxB* gene, which is involved in LPS synthesis, inhibited *A. baumannii* growth (Martínez-Guitián et al. 2020). Similarly, a promising compound named Zosurabalpin (RG6006), which has a completed clinical pharmacokinetic study, adversely affects the growth of extensively drug-resistant strains of CRAB by targeting LPS (Hoffmann-La Roche 2024; Zampaloni et al. 2024).

Furthermore, the antivirulence approach of inhibiting QS was argued to violate the criterion of having a neutral effect on bacterial growth under clinical nutrient-limited conditions, as opposed to the rich media used in the laboratory (García-Contreras et al. 2013). That is because it is likely that inhibiting the complex QS machinery, which is regulated by hundreds of genes, may eventually influence growth under non-experimental conditions (Schuster et al. 2003; Wagner et al. 2003; García-Contreras et al. 2013).

### Narrow spectrum of activity

An optimal antivirulence drug may target a conserved virulence trait that permits its use for a broad spectrum of infections (Rasko and Sperandio 2010). However, this is not the case for narrow-spectrum antivirulence therapeutics, given the differential expression of virulence traits in different clinical isolates (Actis 2010; Mühlen and Dersch 2016). In such cases, rapid diagnostics would be a prerequisite to the use of narrow-spectrum antivirulence drugs (Escaich 2008; Laxminarayan et al. 2013; Mühlen and Dersch 2016; Lau et al. 2023). This is especially important in a pathogen of high genomic plasticity, such as *A. baumannii* (Imperi et al. 2011; Valcek et al. 2023). Rapid diagnostics are becoming routine in clinical practice (Lau et al. 2023). Yet, the applicability of narrow-spectrum antivirulence therapeutics would still be limited in settings where rapid modern diagnostics are not readily available for clinicians to make patient-specific, personalized treatment decisions, such as in low- and middle-income countries (LMICs) (Ombelet et al. 2018; Chamas et al. 2022).

### Cost of development

Collaboration between academia and industry is crucial to circumvent the cost of preclinical and clinical trials, which academic research labs mostly cannot afford (Czaplewski et al. 2016; Maura et al. 2016; Neville and Jia 2019). So that antivirulence therapeutics can be carried further to clinical testing, safety and tolerability assays, as well as formulation studies must be carried out, which requires the industry to get involved with its research and development infrastructure as soon as possible (Mühlen and Dersch 2016).

## Opportunities in antivirulence drug development against *A. baumannii*

### Pool for antivirulence drug discovery

Unconventional research efforts to find antivirulence drugs ought to be ventured. There may be an interesting venue to explore non-bactericidal antivirulence properties in antivirulence agents targeting conserved Gram-negative virulence factors, which are reported to have no in vitro killing activity against *A. baumannii*. This has been the case for LpxC-1, an LPS biosynthesis inhibitor reported earlier. Another pool of possible antivirulence drugs is testing marketed antibiotics at doses lower than the MIC. Repurposing of marketed or clinically tested antibiotics, such as Zosurabalpin, can hasten the antivirulence drug development pipeline. Indeed, sub-inhibitory concentrations of trimethoprim and sulfamethoxazole inhibited biofilm formation in *A. baumannii* (Moon et al. 2017). In addition, testing colistin at a sub-MIC dose as an antivirulence drug adjunct to conventional antibiotics was argued by Lin et al. (2012) to be worth investigating since colistin was found to inhibit LPS-mediated TLR-4 activation in vitro.

Another way to find antivirulence drugs against *A. baumannii* is to carry on with agents with proven in vivo efficacy in other Gram-negative bacterial infections. One such agent is the aforementioned phosphorylated polymer, Pi-PEG 15–20. The polymer was tested against *A. baumannii* in an invertebrate infection model. An ex vivo assembled multi-pathogen community of clinically obtained isolates, including MDR *Serratia marcescens*, MDR *Klebsiella oxytoca*, *Enterococcus faecalis*, and *Candida albicans* was used to induce peritoneal sepsis in male C57BL/6 mice. The Pi-PEG 15–20 enema-treated group was found to be completely protected compared to the control group, which suffered a high mortality rate in the seven-day experiment. Notably, Pi-PEG 15–20 was able to maintain microbiota diversity as evidenced by the taxonomical analysis based on identifying the microbial protein content in the lysate of cecal contents obtained at the point of sacrificing animals (Zaborin et al. 2014). It could be interesting to test Pi-PEG 15–20 in murine *A. baumannii* infection models. Another example is 3,3’-Diindolylmethane (DIM), a cruciferous phytochemical, which had anti-biofilm and biofilm detachment activity against *A. baumannii* and *P. aeruginosa in vitro* without affecting bacterial growth. When tested in vivo using a porcine *P. aeruginosa* wound infection model, DIM could reduce bacterial loads and wound size in a manner superior to the DIM/gentamycin combination-treated group (Golberg et al. 2022). Interestingly, DIM has undergone several clinical trials as a chemopreventive agent (Reyes-Hernández et al. 2023).

### Low resistance development likelihood

The major argument for antivirulence drugs is the expected low selection pressure for resistance development. Even though an ideal antivirulence drug should not affect growth, indirect fitness costs can still arise. Some researchers reported cases of resistance to antivirulence drugs (Allen et al. 2014; Maura et al. 2016). The reported molecular mechanisms of resistance included single-point mutations in the target protein, mutations in the binding site, and mutations that increase drug efflux (Hung et al. 2005; Maeda et al. 2012; Smith et al. 2012). However, we contend that these reports of deliberately generated mutants resistant to antivirulence compounds are not equivalent to the clinical scenario of developing resistance after exposure to an antivirulence drug for a certain period. In some cases, these mutants were rather generated for experimental purposes to validate the mechanism of action (Hung et al. 2005; Koch et al. 2005; Smith et al. 2012). While in others, they aimed to explore the possible mechanisms of resistance (Maeda et al. 2012; Travier et al. 2013). In this regard, we agree with the opinion article by Allen et al. (2014) where they argued that, in the case of antivirulence drugs, the presence of resistance mechanisms may not necessarily translate into their transmission to an alarming level in clinical settings, depending on the contribution of the virulence factor expression to the pathogen fitness. Our understanding of antivirulence resistance development is largely hypothetical due to the lack of clinical data from human or animal testing. However, the selective pressure for resistance to antivirulence agents is widely accepted to be much weaker than for traditional antibiotics, a critical factor that may help preserve their long-term effectiveness.

Early in the development pipeline, researchers can choose virulence targets for which resistance is less likely to develop. Allen et al. (2014) outlined scenarios where an antivirulence drug could select against resistance depending on the nature of the virulence factor targeted, its environment, and the structure of the bacterial community. For example, a virulence factor that qualifies as a potential antivirulence target is preferable to exert beneficial effects distant from the site of infection (Maura et al. 2016). Theoretically, this can be because the energy cost of expressing such a distantly beneficial virulence factor outweighs its benefits, which do not directly contribute to the local infection microenvironment. Similarly, secreted virulence factors offer shared benefits with non-resistance pathogen cells. Thus, resistant cells pay expression costs for “common goods”. Such shared virulence factors are hypothesized to be more readily dispensed by resistant cells in favor of other factors offering “private” benefit (Allen et al. 2014). Moreover, administration of antivirulence drugs can be presumed to select better-adapted pathogen variants (Mühlen and Dersch 2016). Since antivirulence therapeutics are expected to be administered in combination with classical antibiotics, the combined antibiotic/antivirulence can still provide a turnaround to control such better-adapted variants if they appear (Mühlen and Dersch 2016; Calvert et al. 2018; Ogawara 2021). This leads to a room of optimism that careful studies into the role of a proposed antivirulence target in the fitness of a bacterial pathogen versus the cost of its expression can lead to the design of more evolutionarily robust antivirulence drugs (Allen et al. 2014).

### Synergy with conventional antibiotics

Antivirulence drugs will likely be used in combination with traditional antibiotics. For *A. baumannii*, which usually affects immunocompromised patients, an antivirulence/antibiotic combination is quite probable both for clinical trial design and post-approval administration to patients. Thus, antivirulence drugs proving preclinical synergy with antibiotics can be in a more favorable position for advancing to clinical trials. It is promising that most of the agents reported in this review, including mAbs, have exhibited synergy with traditional antibiotics such as colistin, a last-resort antibiotic for CRAB infections.

### Rapid diagnostics

For an antigenically diverse organism such as *A. baumannii*, point-of-care rapid diagnostics should augment narrow-spectrum antivirulence therapeutics. Aiming to achieve universal mAb coverage, a single-step flow cytometry-based binding assay and a mAb-conjugated latex bead agglutination assay were developed to predict the in vivo efficacy of anti-*A. baumannii* mAbs (Slarve et al. 2024). Similarly, the specificity of phage-derived enzymes and the diversity of bacterial capsules will necessitate typing of the bacterial strain in clinical settings before initiating treatment. To overcome their narrow spectrum, it is proposed to use an enzyme cocktail, an approach already used in clinical phage therapy (Chen et al. 2022), and protein engineering schemes are expected to widen the spectrum of activity of depolymerases after the exact mechanisms of polysaccharide cleavage are elucidated (Latka et al. 2017). A panel of clinical *A. baumannii* isolates was designed to reflect the bacterial genetic diversity (Galac et al. 2020). Researchers can use such a panel to develop or assay antivirulence therapeutics with broad strain coverage. Maura et al. (2016) argued that the issue of expanding specificity should be balanced to keep adverse effects on beneficial microbiota to a minimum. The narrow spectrum of activity, on the other hand, can be viewed as an advantage since it would salvage beneficial microbiota and limit selection for resistance (Laxminarayan et al. 2013; Chen et al. 2022).

### Commercial incentives to the pharmaceutical industry

Investment in antivirulence drug development may prove to be a profitable venue for the pharmaceutical industry as opposed to the loss of interest in developing novel antibiotics and the subsequent stagnation of antibiotic development pipelines (Mühlen and Dersch 2016). This review summarized several cases of humanizing mAbs (Nielsen et al. 2017; Nielsen et al. 2021; Slarve et al. 2023). These represent an example of academia-industry partnerships that can strengthen our stance against *A. baumannii* infections while being mutually beneficial to both collaborating parties.

Finally, although we contend that the reported antivirulence approaches are promising for being tested in vivo and exhibiting synergy with conventional antibiotics, it is concerning that no antivirulence agents are in clinical development for *A. baumannii*. The translation of *A. baumannii* antivirulence therapy to clinical studies and authorization is contingent on investment from the industrial sector. According to our investigation, the fact that no antivirulence agents are in clinical development for *A. baumannii* might be attributable to a lack of interest from the pharmaceutical industry in investing in this pathogen. While investments directed to chronic infections can be more profitable, since the pool of patients is likely to use a drug product for a prolonged period, *A. baumannii* infections are, by definition, acute. That said, investment costs in drugs for acute diseases are often offset by the price of the drug product. This, in turn, might create a discrepancy in drug availability between developed and LMICs, the latter being mostly affected by higher resistance rates among *A. baumannii* isolates. It is here that international organizations such as the WHO should work to bridge the gap in equitable access to novel treatment options for *A. baumannii*, especially in cases of outbreaks. Unless the pharmaceutical industry finds it profitable to launch antivirulence pipelines targeting *A. baumannii*, all preclinical drug development efforts might remain untranslated. Overall, it is promising that this review highlights cases of academia-industry collaborations, exemplified in mAb development. We are optimistic that such collaborations can be replicated in other antivirulence approaches to strengthen our stance against *A. baumannii*.

## Summary

**Fig. 1:**
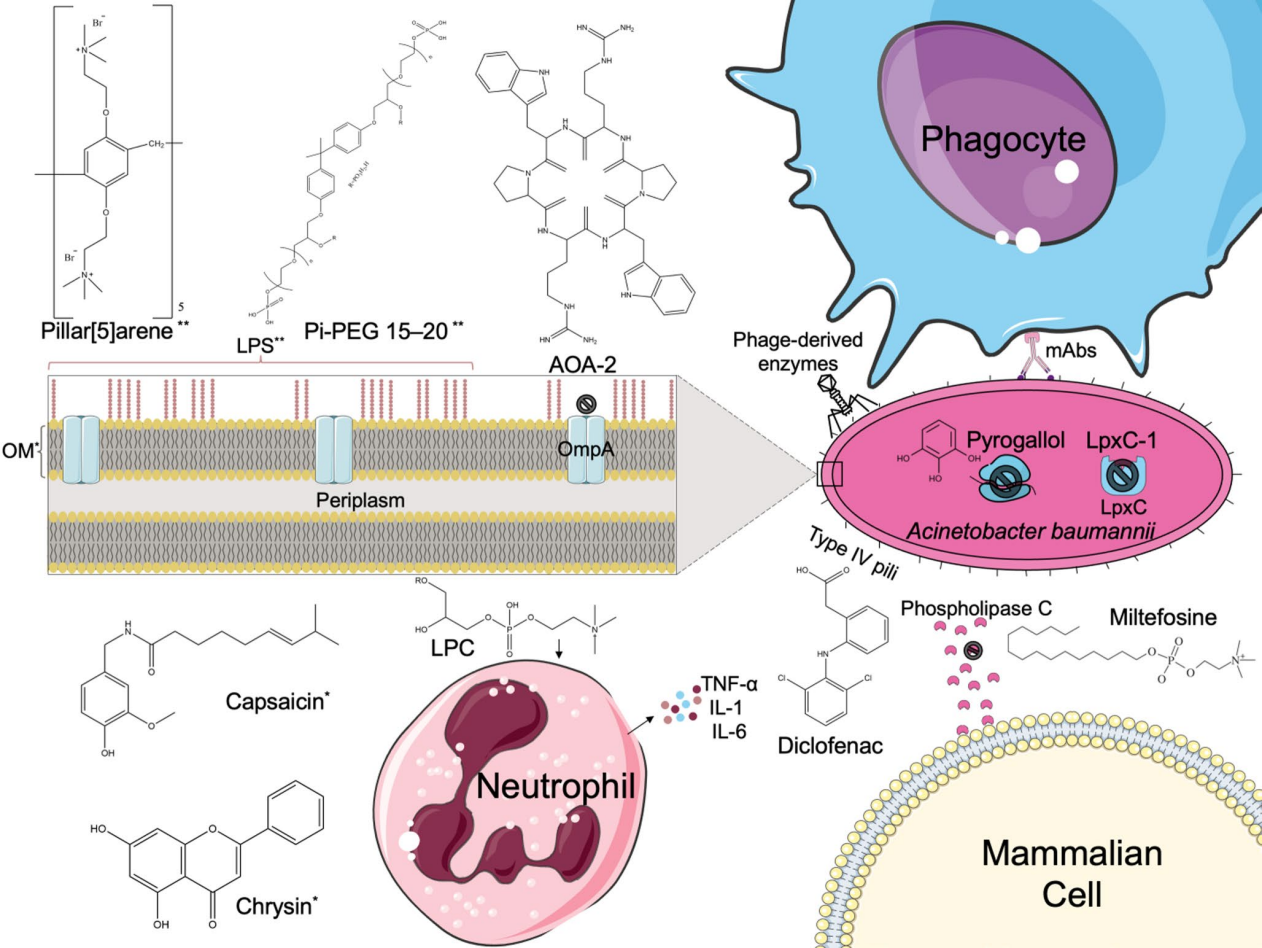
Summary of the reported *A. baumannii* antivirulence therapeutics and their targets. IL-1, interleukin 1; IL-6, interleukin 6; LPC, lysophosphatidylcholine; LPS, lipopolysaccharide; LpxC, UDP-3-O-(R-3-hydroxymyristoyl)-N-acetylglucosamine deacetylase; mAb, monoclonal antibody; OM, outer membrane; OmpA, outer membrane protein A; Pi-PEG 15–20, Phosphorylated high-molecular-weight polyethylene glycol 15–20; TNF-α, tumor necrosis factor alpha; ^*^ antivirulence agents targeting OM; ^**^ antivirulence agents targeting LPS. The icons used in Fig. 1 were obtained from Bioicons and Flaticon, then adapted as needed under licenses CC-BY 4.0 Unported and CC-BY 3.0 Unported

Targeting virulence factors represents a transformative approach to addressing resistant pathogens without directly threatening bacterial survival, thereby lowering selection for resistance. Given the multitude of studies on *A. baumannii* virulence factors, translating these studies into antivirulence therapeutics still seems to lag. Yet, we find the agents reported here and summarized according to their targets in Fig. 1 to be promising. They exert their effects through diverse mechanisms aimed at disarming *A. baumannii*. These include the disruption of the bacterial capsule, a critical determinant of immune evasion and persistence; interference with LPS synthesis, a cornerstone of outer membrane stability; and targeting outer membrane proteins or specific metabolic pathways integral to pathogenicity. Several agents exploit unique host-pathogen interactions to impair immune evasion strategies, neutralize virulence-related surface antigens, or potentiate host immune responses.

## Conclusion

We argue that all the agents reported here have attributes supporting their clinical translation. All the agents were tested in vivo and mostly in vertebrate models. It is also promising that most of the approaches reported were synergistic with conventional antibiotics. Besides, their effectiveness against clinical *A. baumannii* strains, spanning XDR, colistin-resistant, and MDR profiles, underscores the translational potential of these approaches. We speculate that for these translational attributes, pharmaceutical industry entities focusing on anti-infectious agent development can consider investing in the *A. baumannii* antivirulence therapeutics reported here.

In particular, immunotherapeutics hold great potential. Several anti-*A. baumannii* mAbs have been developed to achieve universal strain coverage with a mAb cocktail. Even though indirectly, LPS synthesis inhibitors can also be thought of as immune modulators, given the ramifications of LPS in systemic inflammation and sepsis. In human hosts, the pre-clinical efficacy of immunomodulating drugs can be replicated by combining them with conventional antibiotics. This augments the promise of immunotherapeutics in combating *A. baumannii* infection.

In addition to the antivirulence approaches reported here, other options can be explored. By investigating antivirulence therapeutics that are currently marketed or in advanced development stages, researchers can hasten antivirulence drug development against *A. baumannii*. This can be especially applicable to antivirulence drugs targeting conserved Gram-negative virulence factors. In addition, screening FDA-approved drugs for antivirulence properties may cut substantial drug development costs and increase antivirulence treatment options for *A. baumannii*. The case of diclofenac reported in this review represents such an example.

We acknowledge the limitations of our current work. We have focused primarily on PubMed and Google Scholar databases. However, we have double-checked that all relevant papers are included for this narrative review. Moreover, we have excluded certain types of anti-virulence drugs, such as anti-biofilm and anti-quorum-sensing, since they are already extensively reviewed in the literature. Accordingly, we have covered 21 in vivo-tested antivirulence therapeutic approaches targeting *A. baumannii* through different mechanisms and thoroughly discussed the challenges and opportunities of *A. baumannii* antivirulence drug development, providing our insights into their current status. This review provides an informative reference for researchers in either academia or industry that can be used to assess where we stand in the fight against *A. baumannii* through novel antivirulence agents.

## Data Availability

No datasets were generated or analysed during the current study.
